# Increased Incidence of Visual Field Abnormalities as Determined by Frequency Doubling Technology Perimetry in High Computer Users Among Japanese Workers: A Retrospective Cohort Study

**DOI:** 10.2188/jea.JE20170004

**Published:** 2018-04-05

**Authors:** Tadashi Nakano, Takeshi Hayashi, Toru Nakagawa, Toru Honda, Satoshi Owada, Hitoshi Endo, Masayuki Tatemichi

**Affiliations:** 1 Department of Ophthalmology, The Jikei University School of Medicine, Tokyo, Japan; 2 Hitachi Health Care Center, Ibaraki, Japan; 3 Department of Preventive Medicine, Tokai University, School of Medicine, Kanagawa, Japan

**Keywords:** computer use, visual field abnormality, FDT

## Abstract

**Background:**

This retrospective cohort study primarily aimed to investigate the possible association of computer use with visual field abnormalities (VFA) among Japanese workers.

**Methods:**

The study included 2,377 workers (mean age 45.7 [standard deviation, 8.3] years; 2,229 men and 148 women) who initially exhibited no VFA during frequency doubling technology perimetry (FDT) testing. Subjects then underwent annual follow-up FDT testing for 7 years, and VFA were determined using a FDT-test protocol (FDT-VFA). Subjects with FDT-VFA were examined by ophthalmologists. Baseline data about the mean duration of computer use during a 5-year period and refractive errors were obtained via self-administered questionnaire and evaluations for refractive errors (use of eyeglasses or contact lenses), respectively.

**Results:**

A Cox proportional hazard analysis demonstrated that heavy computer users (>8 hr/day) had a significantly increased risk of FDT-VFA (hazard ratio [HR] 2.85; 95% confidence interval [CI], 1.26–6.48) relative to light users (<4 hr/day), and this association was strengthened among subjects with refractive errors (HR 4.48; 95% CI, 1.87–10.74). The computer usage history also significantly correlated with FDT-VFA among subject with refractive errors (*P* < 0.05), and 73.1% of subjects with FDT-VFA and refractive errors were diagnosed with glaucoma or ocular hypertension.

**Conclusions:**

The incidence of FDT-VFA appears to be increased among Japanese workers who are heavy computer users, particularly if they have refractive errors. Further investigations of epidemiology and causality are warranted.

## INTRODUCTION

The rapid global spread of information technology (IT), and most recently of new media (eg, smartphones and tablets), has continually increased exposure to visual display terminals (VDT). This exposure is particularly marked among general workers, who are exposed in both the workplace and during private activities. These backlit VDT apparatuses, such as monitors, expose the eyes to direct light stimulation for long periods of time. In addition to ophthalmic issues, long-term VDT use (ie, computer use) could lead to a reduction in physical activities, sleep disturbances, or life rhythm disorders that result in mental and/or physical impairments.^1^^–^^3^ Accordingly, the health issues related to long-time computer use are of great concern.^4^^,^^5^

Glaucoma is a relatively common age-related ocular disease characterized by optic nerve degeneration for which the precise etiology is largely unknown.^6^ Many epidemiological studies of glaucoma have identified that the characteristics, prevalence, and type of glaucoma depend on race.^6^^,^^7^ In Japan, primary open-angle glaucoma is the dominant form of this condition, and normal tension glaucoma accounts for >90% of primary open-angle glaucoma cases.^8^ Myopia has been generally suggested as a strong risk factor for glaucoma both in general^9^ and in the Japanese population,^10^^,^^11^ and smoking,^12^ regular short sleep,^13^ systemic hypoxia (including sleep apnea syndrome),^14^ hypertension,^15^ obesity, and diabetes^16^ have also been suggested. We postulated that long-time computer use might increase the risk of glaucoma.

A glaucoma screening method was needed to investigate this risk, as affected patients do not exhibit subjective visual field losses until they reach the terminal stage.^6^ Initially, we studied the effectiveness of frequency doubling technology perimetry (FDT) for mass glaucoma screening.^17^ FDT-based visual field testing is advantageous for mass screening because it is simple, of short duration, portable, and does not require refraction correction or expert technicians. Our initial studies of the applicability of FDT-based mass screening^17^^,^^18^ revealed that >70% of identified visual field abnormalities (VFA) correlated with glaucoma.^17^ Accordingly, we set the FDT test findings as an outcome of the present study.

Our previous large-scale, population-based study of FDT in the workplace indicated a possible association between a history of computer use and glaucoma.^11^ Interestingly, we observed a significant interaction effect of myopia on the association between computer use and glaucoma; specifically, an increased risk of glaucomatous VFA was observed only among frequent computer users with myopia.^11^ Our recent study confirmed this finding in different population.^19^ However, those studies featured a cross-sectional design. To obtain more precise evidence, we designed a retrospective cohort study to investigate the association between computer use and VFA using the FDT test.

## MATERIALS AND METHODS

### Study subjects

This study was approved by the Ethics Committee of Showa University and conducted at a health center belonging to a member of a group of large-scale electronics companies. Employees and their spouses (≥39 years of age) from 35 affiliated companies (38,000 employees) freely selected the timing and health center where they would undergo health examinations. Subjects were recruited from among all 4,184 participants who underwent examinations at this health center for 3 months from 2000 through 2001 and were employed mainly at two companies associated with research and development (7,000 total employees). Of the individuals examined during the study period, 2,806 (mean age: 46.1 [standard deviation {SD}, 8.5] years; male:female ratio: 2,623:183) initially underwent FDT (Carl Zeiss Meditec, Oberkochen, Germany) testing (screening mode C-20-1) and responded completely to questions regarding computer use. Of these, 182 exhibiting VFA on FDT testing (FDT-VFA), with unreliable results, or with existing glaucoma were excluded. After another 247 subjects dropped out during the first year, the study cohort comprised 2,377 subjects (mean age: 45.7 [SD, 8.3] years; male:female ratio: 2,229:148). FDT testing was conducted annually for 7 years.

### FDT-VFA detection protocol

The FDT testing protocol used to detect FDT-VFA has been described in detail elsewhere.^17^ Briefly, the protocol comprised two algorithms: reproducibility and decision. Reproducibility was determined via immediate retest after the detection of any VFA during the initial FDT test, and a positive result was noted if a VFA ascertained during the retest was the same as or close to that identified during the initial test. The decision algorithm was considered positive when the FDT results revealed one or more VFAs with mild relative losses located within four central spots on the nasal side of the eye, two or more VFAs in any location, or one or more VFAs with a moderate or severe relative loss in any location.

At the baseline, all participants completed a self-administrated questionnaire about their private lifestyles and working habits, including existing ocular hypertension and glaucoma, smoking habits, and computer use. Nurses obtained information about refractive errors by checking for the presence of such errors (ie, use of eyeglasses or contact lenses) based on subjects’ self-reports. Subjects with FDT-VFA underwent complete ophthalmic examinations by ophthalmologists, and diagnostic information was obtained from hospitals.

### Classification of computer use

For data analysis, we classified computer use using two parameters: 1) mean daily time spent at a computer during the past 5 years and 2) history of computer use. The mean daily time spent at a computer during the past 5 years was divided into four categories and scored as follows: <1 hour, 1; 1–4 hours, 2; 4–8 hours, 3; and >8 hours, 4. The history of computer use was evaluated using the computer use index (CUI).^11^ First, subjects were divided into four categories based on their responses, and scores were assigned as follows: <5 years, 1; 5–10 years, 2; 10–20 years, 3; and >20 years, 4. We then established a CUI using the following formula:

CUI = score for history of computer use score × score for mean daily time spent at the computer over 5 years.

Subjects were then classified by CUI as follows: 1–3, light users; 4–8, moderate users; and 9–16, heavy users.

### Follow-up

Subjects participated in annual follow-up FDT testing during health examinations according to the above-described FDT detection protocol.

### Statistical analysis

The chi-squared test or one-way analysis of variance, followed by Bonferroni’s multiple comparison test, was used to determine statistical significance among the three CUI groups. If a subject had not undergone FDT testing during a particular year but received a normal result during the following year, a normal result was recorded for both years. An event was defined as a newly identified FDT-VFA. A case was censored if FDT testing had not been performed following the last normal FDT test or if no FDT-VFA had been identified within 7 years after the baseline.

As none of the variables selected for the final model had a *P*-value of <0.05 in the Schoenfeld residuals test (eg, Schoenfeld residuals test for computer use, *P* = 0.773), there was no violation of the assumption of the Cox proportional hazards model. Potential confounding variables including age (years), sex (man or woman), body mass index (BMI) (kg/m^2^), systolic blood pressure (mm Hg), existing ocular hypertension (present or not), family history of glaucoma (present or not), and smoking status (never/former or current) were selected based on our previous cross-sectional study.^11^ First, the Cox proportional hazards model was used to calculate the hazard ratios (HRs) and 95% confidence intervals (CIs) of possible confounding variables, refractive errors, and computer use. Second, we investigated the association between computer use and FDT-VFA after stratification by refractive errors, given our previous observation of the potent interaction effect of refractive errors on the association between computer use and FDT-VFA^11^^,^^19^ and the significant correlation of computer use and refractive errors (*P* < 0.001).

Among subjects with refractive errors, the Kaplan-Meier method was used to analyze the probability of FDT-VFA accumulation, and significance differences by computer use status were determined using the log-rank test. All statistical analyses were performed using IBM-SPSS^®^ version 22.0 (IBM SPSS^®^, Tokyo, Japan) except for the Schoenfeld residuals test, which was conducted using SAS version 9.2 (SAS Institute Japan Ltd, Tokyo, Japan). Mean values were presented with standard deviations. Values were considered statistically significant at *P* < 0.05.

## RESULTS

The baseline characteristics of the subjects are summarized in Table 1. Notably, we observed significant differences in age, sex, BMI, and refractive errors among the three groups stratified by the mean time spent at a computer during a 5-year period and CUI. For example, light users were significantly older than moderate or heavy users. The mean follow-up duration was 5.8 (SD, 2.0) years (including 432 subjects who skipped the annual FDT test). During a total of 13,975 person-years, new FDT-VFA occurred in 94 cases, yielding an incidence rate of 6.7/1,000 person-years.

**Table 1.** Characteristics of study subjects

Variables		5-year mean time spent at a computer			*P*	Computer use index			*P*	Total
	
		<4 hr/day	4 hr–8 hr/day	>8 hr/day		1–3	4–8	9–		
		
		*n* = 1,529	*n* = 759	*n* = 89		*n* = 1,082	*n* = 1,036	*n* = 259		*n* = 2,377
Age	mean (SD)	47.3 (8.5)	42.9 (6.9)	41.9 (7.3)	<0.001	47.6 (8.7)	44.8 (7.8)	41.2 (5.7)	<0.001	45.7 (8.3)
Sex	men, %	92.6	95.7	97.8	0.005	90.0	96.9	96.9	<0.001	93.8
BMI	mean (SD)	23.1 (2.8)	23.5 (3.0)	23.5 (3.2)	0.003	22.9 (2.8)	23.4 (2.9)	23.8 (3.1)	<0.001	23.3 (2.9)
Systolic blood pressure	mean (SD)	121.4 (13.5)	120.8 (13.0)	120.0 (11.7)	0.429	121.6 (13.4)	120.6 (13.3)	121.1 (13.0)	0.246	121.1 (13.3)
Ocular hypertension	present, %	4.3	4.3	1.1	0.340	4.7	3.4	5.1	0.256	4.2
Family history of glaucoma	present, %	6.7	6.1	5.6	0.810	6.2	7.5	3.9	0.103	6.5
Smoking status	current smoker, %	21.7	21.3	16.9	0.553	22.1	21.1	19.7	0.672	21.4
Refractive errors	present, %	47.0	68.4	70.8	<0.001	42.1	63.7	71.0	<0.001	54.7

BMI, body mass index; SD, standard deviation.

Information on refractive errors was obtained by checking for the presence of refractive errors (use of eyeglasses or contact lenses) by nurses on the basis of a self-report.

In this study, age, existing ocular hypertension, smoking status, refractive errors, and mean time spent using a computer were listed as significant variables (Table 2). In a univariate analysis, age was positively associated with FDT-VFA, and existing ocular hypertension, current smoking, present refractive errors, and long-time computer use appeared to be positively associated with FDT-VFA. Next, a multiple-adjusted hazard ratio was calculated using all listed variables. Consequently, age, existing ocular hypertension, and computer use remained significant. The hazard ratio of long-time (>8 hr/day) computer use was 2.85 (95% CI, 1.26–6.48), indicating a significantly positive association with FDT-VFA.

**Table 2.** Univariate and multivariate analysis of the risk factors for FDT-VFA

Variables		FDTVFA(+)	Crude				Multiple-adjusted^a^			
	
			Hazard ratio	95% Cl		*P*	Hazard ratio	95% CI		*P*
Age	mean (SD)	47.6 (8.7)	1.04	1.01	1.06	0.004	1.04	1.01	1.06	0.016
Sex	man (*n* = 2,229)	4.1%	reference				reference			
	woman (*n* = 148)	2.0%	0.53	0.17	1.68	0.280	0.79	0.24	2.49	0.658
BMI	mean (SD)	23.6 (3.3)	1.04	0.98	1.12	0.215	1.04	0.97	1.12	0.312
Systolic blood pressure	mean (SD)	122.1 (13.7)	1.01	0.99	1.02	0.407	1.00	0.98	1.02	0.821
Ocular hypertension	not (*n* = 2,250)	3.5%	reference				reference			
	present (*n* = 98)	11.2%	3.36	1.79	6.32	<0.001	2.79	1.42	5.48	0.003
Family history of glaucoma	not (*n* = 2,174)	3.9%	reference				reference			
	present (*n* = 98)	4.0%	1.02	0.46	2.33	0.965	0.84	0.34	2.08	0.706
Smoking-status	never or former (*n* = 1,868)	3.5%	reference				reference			
	current (*n* = 509)	5.5%	1.56	1.02	2.47	0.041	1.27	0.78	2.07	0.332
Refractive error	not (*n* = 1,077)	3.1%	reference				reference			
	present (*n* = 1,300)	4.7%	1.53	1.00	2.34	0.048	1.43	0.90	2.24	0.127
Computer use										
	<4 h/day (*n* = 1,529)	3.3%	reference				reference			
	4–8 h/day (*n* = 759)	4.7%	1.39	0.91	2.13	0.129	1.45	0.90	2.33	0.126
	>8 h/day (*n* = 89)	7.9%	2.36	1.07	5.19	0.033	2.85	1.26	6.48	0.012

BMI, body mass index; CI, confidence interval; FDT-VFA, visual field abnormalities determined using an FDT-based protocol; SD, standard deviation.

^a^Adjusted for all listed variables by Cox-proportional hazard model.

Table 3 lists the potential confounding factor-adjusted hazard ratios and 95% CIs for the mean time spent at a computer during a 5-year period, stratified by the presence of refractive errors. Among subjects with refractive errors, we observed a significantly positive association between the mean time spent at a computer and FDT-VFA (*P* for trend = 0.002). A heavy computer user status (>8 hr/day) was associated with a significantly increased risk of FDT-VFA (HR 4.48; 95% CI, 1.87–10.74). Further analysis revealed a significantly positive association between CUI and FDT-VFA among subjects with refractive errors (HR 2.46; 95% CI, 1.26–4.81 for moderate users; HR 2.43; 95% CI, 0.97–6.06 for heavy users; *P* for trend = 0.021; Table 3). In contrast, no association between the time spent at a computer and FDT-VFA was observed among subjects without refractive errors (HR 0.84; 95% CI, 0.34–2.10 for moderate users and HR 0.00 for heavy users; *P* = 0.998). A similar lack of association was observed for CUI among subjects without refractive errors (HR 0.88; 95% CI, 0.40–1.95 for moderate users and HR 0.91; 95% CI, 0.20–4.15 for heavy users).

**Table 3.** Adjusted hazard ratios and 95% confidence intervals for the association of computer use with the FDT-VFA among subjects with refractive errors

		Multiple-adjusted^a^ hazard ratio	95% CI		*P*
5-year mean time spent at a computer at the base-line	<4 hr/day	1	reference		
	4 hr–8 hr/day	1.96	1.09	3.56	0.025
	>8 hr/day	4.48	1.87	10.74	0.010

	*P* for trend = 0.002				

	Computer use index				
History of computer use at the base-line	1–3	1	reference		
	4–8	2.46	1.26	4.81	0.009
	9–	2.43	0.97	6.06	0.057

	*P* for trend = 0.021				

CI, confidence interval; FDT-VFA, visual field abnormalities determined using an FDT-based protocol.

^a^Adjusted for age, sex, BMI, systolic blood pressure, ocular hypertension, family history of glaucoma, and smoking status.

**Figure 1.**
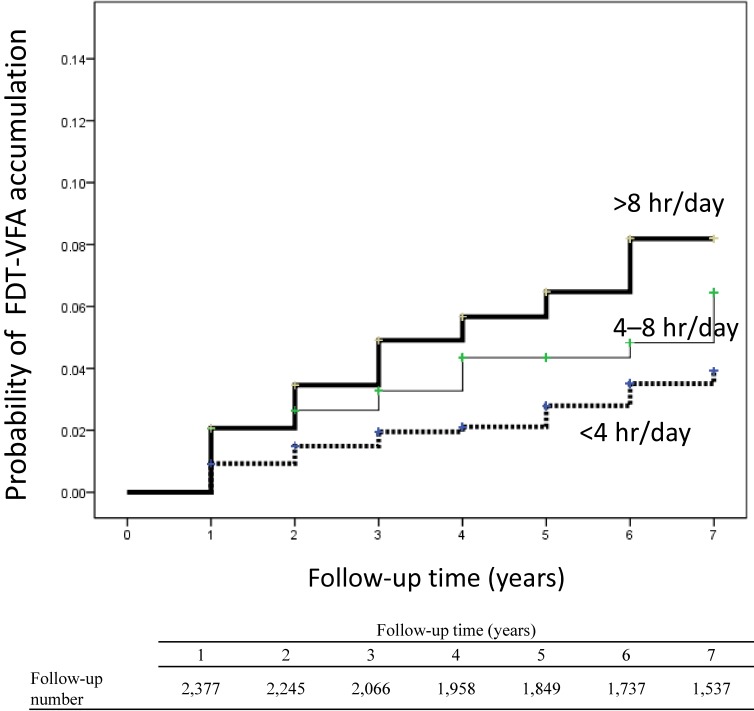
Probability of visual field abnormality accumulation determined using the FDT test and stratified by the 5-year mean time spent at a computer. The probability of visual field abnormality accumulation, determined using the FDT test, was calculated using the Kaplan-Meier method. Bold, solid, and broken lines indicate 5-year mean times spent at a computer of >8 hr/day, 4–8 hr/day, and <4 hr/day, respectively. FDT, frequency doubling technology perimetry.

Among subjects with refractive errors, a Kaplan-Meier analysis showed that the probability of FDT-VFA accumulation was higher among heavy users (log-rank test, *P* = 0.047) (Figure 1).

We were able to obtain diagnostic information for 41 of the 61 subjects with FDT-VFA and refractive errors from hospitals. Of these, 26 (63.4%) were diagnosed with glaucoma, 4 (9.7%) with ocular hypertension, 4 (9.7%) with severe myopia, 2 (4.9%) with retinal detachment, 2 (4.9%) with other diseases, and 3 (7.3%) as normal.

## DISCUSSION

To our knowledge, this was the first study in which a large number of subjects underwent several rounds of follow-up perimetric testing to investigate the association between computer use and VFA. Our results showed that heavy computer users, particularly those with refractive errors, had a significantly increased incidence of FDT-VFA and that glaucoma was the most common ophthalmological diagnosis in those participants for whom diagnostic information was available from hospitals. These findings were similar to previous results from our cross-sectional studies.^11^^,^^19^

Our previous studies demonstrated that myopia was the underlying form of refractive error among the majority of Japanese workers.^17^^–^^20^ However, the links among myopia, heavy computer use, and glaucoma susceptibility remain largely unknown. Regarding pathological mechanisms, shearing forces toward the long axis in the myopic eye ball might cause structural weakness of the optic disc and microcirculatory disturbances, leading to glaucoma.^21^ A recent study found that myopia might worsen glaucomatous visual field impairment via myopic optic disk deformation, rather than directly via refractive error or axial length.^22^ In addition to this glaucoma-susceptible eye condition, long-time computer use might interactively increase the risk of glaucoma. Myopia might also be associated with a bias toward educational factors that lead to differences in lifestyles or working habits, as intelligence and education appear to be important triggers of this condition.^23^^–^^25^ The lifestyles and working habits of heavy computer users with myopia might introduce possible risk factors for glaucoma.

In this study, the current computer usage (ie, 5-year mean time spent at a computer) was more strongly associated with FDT-VFA than was the history of computer use (evaluated via the CUI), whereas our previous cross-sectional study revealed the opposite association pattern. Our current data indicate that long daily usage time might have a stronger effect on FDT-VFA development than long-term use. We also note that long sitting duration might affect the onset of glaucoma. Long daily time at a computer may be more closely associated with long daily sitting time than long-term computer usage per se. A recent systematic review described an increase in oxidative stress among glaucoma patients,^26^ and reactive oxygen metabolites were observed at higher levels in sitting subjects than standing subjects.^27^

This study included several limitations. First, we did not stratify the ophthalmologically determined spherical diopter powers of subjects by computer use. Heavy computer users might have a more severe grade of myopia than light users. Therefore, we cannot rule out selection bias. Second, unadjusted confounding factors, such as a decreased sleep duration and low physical activity level, might affect the risk of glaucoma.^28^ Third, we only assessed computer use at baseline, and usage patterns might have changed during the follow-up period. Therefore, more precise studies of ophthalmological findings and lifestyles are needed to address these limitations.

Despite these limitations, our data indicate that heavy computer users, and particularly those with myopia, should undergo regular visual field testing. The recent increases in myopia prevalence^23^^,^^29^^,^^30^ and computer usage coincide with the IT revolution. Accordingly, glaucoma might become an important global public health issue in the future.
